# Densitometry and temperature measurement of combustion gas by X-ray Compton scattering

**DOI:** 10.1107/S1600577516001740

**Published:** 2016-02-17

**Authors:** Hiroshi Sakurai, Nobuyuki Kawahara, Masayoshi Itou, Eiji Tomita, Kosuke Suzuki, Yoshiharu Sakurai

**Affiliations:** a Department of Electronics and Informatics, Gunma University, 1-5-1 Tenjin-cho, Kiryu, Gunma 376-8515, Japan; b Department of Mechanical and Systems Engineering, Okayama University, Tsushima-Naka 3, Okayama 700-8530, Japan; c Japan Synchrotron Radiation Research Institute (JASRI), 1-1-1 Kouto, Sayo-cho, Sayo-gun, Hyogo 679-5198, Japan

**Keywords:** Compton scattering, laminar flame, imaging, temperature, combustion reaction

## Abstract

Measurement of combustion gas by high-energy X-ray Compton scattering is reported. The intensity of Compton-scattered X-rays has shown a position dependence across the flame of the combustion gas, allowing us to estimate the temperature distribution of the combustion flame. The energy spectra of Compton-scattered X-rays have revealed a significant difference across the combustion reaction zone, which enables us to detect the combustion reaction. These results demonstrate that high-energy X-ray Compton scattering can be employed as an *in situ* technique to probe inside a combustion reaction.

## Abstract 2

Measurement of combustion gas by high-energy X-ray Compton scattering is reported.

## 1. Introduction   

Recent automobile technology requires measurement of complex combustion phenomena in order to achieve cleaner exhaust and less carbon dioxide emission. The combustion occurs due to complex interactions involving heat, mass and momentum transfer. The local flow temperature is a key consequence of the complex phenomena and must be accurately measured. Non-intrusive temperature measurements offer important advantages in combusting flows since they do not introduce disturbances that could alter the flow characteristics and change the temperature distribution (Xiao *et al.*, 2000 ▸). Holographic temperature measurements have been suggested to accurately determine the refractive index in flames in order to infer the temperature distribution (Tieng *et al.*, 1992 ▸; Xiao *et al.*, 2000 ▸). However, these measurements are sensitive to mechanical noise, and hence it is not easy to obtain accurate measurements in a combustion instrument.

High-energy X-ray Compton scattering can provide a non-intrusive measurement of the temperature distribution of the combustion gas in a flame with high accuracy and robustness, because it does not have the drawbacks of the holographic measurements. The intensity of Compton-scattered X-rays for monochromatic X-ray beams, d*N*, is given by
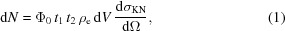
where Φ_0_ is the incident photon flux into a target object, *t*_1_ is the incident X-ray transmittance to a probing volume in the object, *t*_2_ is the scattered X-ray transmittance from the probing volume to an X-ray detector, ρ_e_ is the average electron density over the probing volume d*V*, and dσ_KN_/dΩ is the Klein–Nishina differential cross section (Sharaf, 2001 ▸). In the case of light-element gases, *t*_1_ and *t*_2_ are treated as constants to a good approximation. Therefore the intensity of Compton-scattered X-rays, d*N*, probes the electron density ρ_e_. The ratio of molecular density to electron density, α, is defined by the following equation,
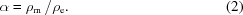
Here ρ_m_ denotes the molecular density. Since the flame system can be expressed by the ideal gas law (Tieng *et al.*, 1992 ▸), we can estimate the temperature distribution from the electron density as follows, 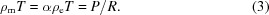
Here, ρ_m_ = *n*/*V* denotes the molecular density, *T* the temperature and *R* the ideal gas constant. The ratio of the molecular density to the electron density, α, can be obtained assuming a combustion reaction. Therefore the intensity of Compton-scattered X-rays gives a temperature *T* if we regard the pressure *P* as a constant value of the ambient pressure, for example.

Furthermore, energy spectra of Compton-scattered X-rays give an electron momentum distribution, which is known as the Compton profile, *J*(*p*_*z*_), given as
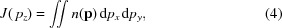


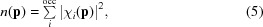


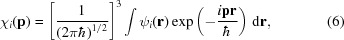


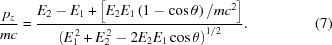
Here, **p** [= (*p*_*x*_, *p*_*y*_, *p*_*z*_)] is the momentum of an electron in a target material. *E*_1_ denotes an incident X-ray energy, *E*_2_ a Compton-scattered X-ray energy with a scattering angle θ, *m* the electron mass and *c* the velocity of light. Since the molecules are not oriented in a certain direction, we can regard *p*_*z*_ = *p*_*x*_ = *p*_*y*_. Electron momentum density is denoted by *n*(**p**). ψ_*i*_(**r**) denotes the wavefunction of the *i*th electronic state under the impulse approximation and hence χ_*i*_(**p**) is the wavefunction of *i*th electronic state in momentum space. Therefore, X-ray Compton scattering provides information on a wavefunction, in other words, chemical bonds and molecular species, through the measurement of electron momentum distribution (Cooper, 1985 ▸). Recently, Itou *et al.* (2015 ▸) have applied this method to a discharging coin cell and successfully detected the change in the local electron density due to lithium ion insertion in the positive electrode.

Despite the merit of a non-intrusive measurement due to the large penetration of X-rays used in the X-ray Compton scattering technique, there are few reports of studies on combustion or flames. This is because Compton scattering from the gas phase is weak. However, the recent availability of high-energy and brilliant synchrotron radiation X-rays have made it possible to perform accurate measurements of Compton-scattered X-rays from low-density materials such as rare gases and N_2_ in ambient atmosphere (Sakurai *et al.*, 2011 ▸; Kobayashi *et al.*, 2011 ▸). In this study, we report the development of a densitometry and temperature measurement technique for use in flames by using Compton scattering.

## 2. Experiment   

The experiment was performed at the BL08W beamline (Sakurai, 1998 ▸) at SPring-8. The X-ray source is an elliptical multipole wiggler operating in the linear polarization mode (Maréchal *et al.*, 1998 ▸). The synchrotron radiation is monochromated and horizontally focused by an asymmetric Johann-type Si monochromator with (620) reflecting planes (Yamaoka *et al.*, 2000 ▸) to deliver 115.6 keV X-ray beams. The entrance slit system defines the size of the incident X-rays to be 1 mm in height and 1 mm in width. Scattered X-rays from the combustion gas in the probing volume were detected by a Ge solid state detector (Ge-SSD) with a collimating silt of 1 mm at a scattering angle of 124.9°. Therefore the probing volume was about 1 mm^3^. The intensity of the incident X-ray beams was monitored by an ion chamber for data normalization. An intensity map of the Compton scattered X-rays was measured for 10 s with 1 mm steps. The Compton scattered X-ray intensity map was measured within 2.5 h.

A cylindrical Bunsen burner (inner diameter *d*_in_ = 10 mm) was used to provide a laminar mixture of propane (6 wt%, 450 ml min^−1^) and air (94 wt%, 8 l min^−1^). The burner provided a laminar propane/air jet at the exit. The air and combustible gases consisted of pure synthetic gases. The temperature distribution was confirmed by a micro-thermocouple probe consisting of a Pt wire butt-welded with Pt-13% Rh wire. The wires were coated by SiO_2_ to avoid catalytic effects. The diameter of the micro-thermocouple probe was 0.5 mm. The burner was mounted on a movable stage, and the intensity of the Compton scattered X-rays from the probing volume was measured by scanning the Bunsen burner along the vertical (*y*) direction and horizontal (*x*) direction on a plane perpendicular to the incident X-ray beam direction.

## 3. Results and discussion   

**Figure 1:**
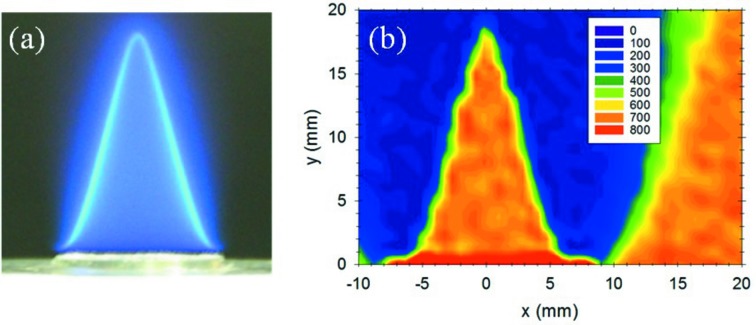
(*a*) Photograph of a self-sustaining flame. (*b*) Cross-sectional map of Compton-scattered X-ray intensities for the flame shown in (*a*). The intensities of Compton-scattered X-rays are shown by the graduated color scale. The orange region shows stronger X-ray intensity and the blue region shows weaker X-ray intensity. The flame center is at *x* = 0 mm.

Fig. 1(*a*) ▸ shows a side-view photograph of the self-sustaining flame. The bright flame front, at which CH* and OH* radicals emit light, shows a cone shape. Fig. 1(*b*) ▸ shows a cross-sectional map of Compton scattered X-ray intensities for the flame shown in Fig. 1(*a*) ▸. The relative intensities of Compton-scattered X-rays are shown by a graduated color scale. The orange region shows strong X-ray intensities and the blue region shows weak intensities. The two sides of the orange triangle region correspond to the bright flame front in Fig. 1(*a*) ▸ and thus the cross-sectional map reproduces the shape of the flame. Since the intensity of the Compton-scattered X-rays has a linear relationship with the electron density averaged over the probing volume of 1 mm^3^ [see equation (1)], the orange region inside the flame cone has a relatively higher electron density and the blue region outside the cone has a relatively lower electron density.

**Figure 2:**
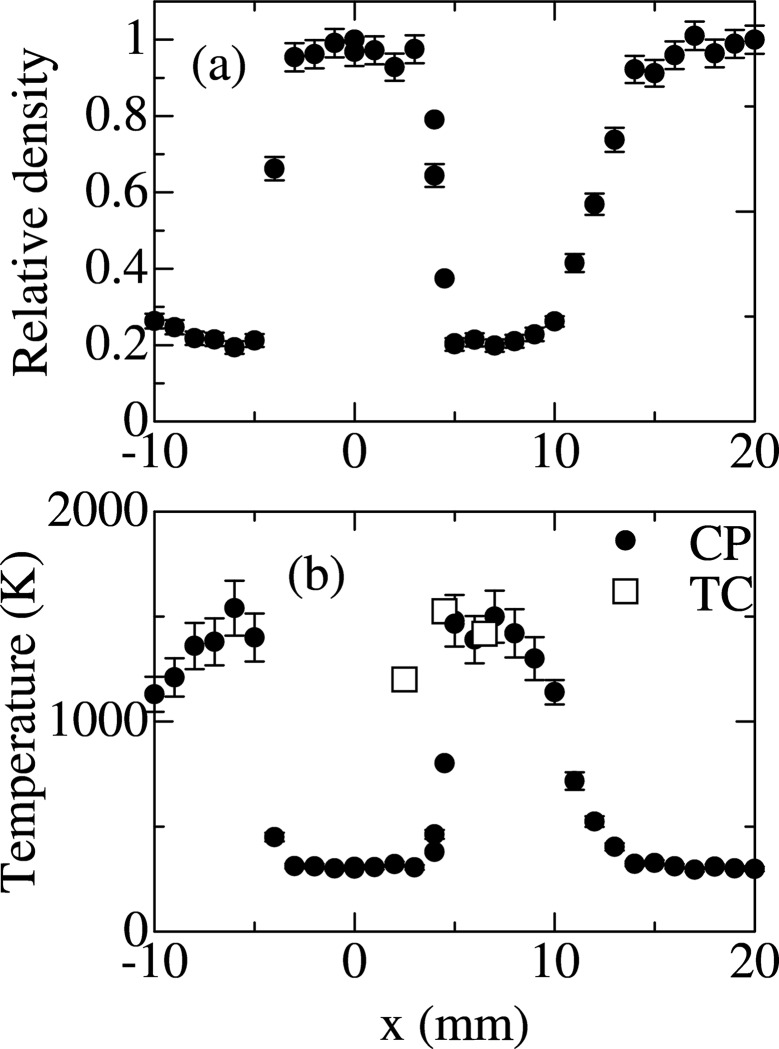
(*a*) Relative electron densities, in which the densities are normalized to that of ambient air, *x* = 20 mm. (*b*) Estimated temperature distribution. The *x* dependences are measured at the position *y* = 5 mm. The positions *x* and *y* are defined in Fig. 1(*b*) ▸. Closed circles denote the temperature by the present Compton scattering measurements (CP) and open squares by thermocouple measurements (TC). The combustion reaction zone is at |*x*| = 4.5 mm.

Fig. 2(*a*) ▸ shows the relative electron densities at the position of *y* = 5 mm and −10 mm < *x* < 20 mm shown in Fig. 1(*b*) ▸. Here the electron densities are normalized to that of the ambient air at *x* = 20 mm. The electron density shows a sudden decrease at the combustion reaction zone of |*x*| = 4.5 mm. The electron density remains almost constant in the inner flame region, |*x*| < 4 mm, which is shown as the orange region in Fig. 1(*b*) ▸. The electron density is one-fifth of the ambient air just in the outer flame region, |*x*| = 5 mm, which is shown as the blue region in Fig. 1(*b*) ▸.

We assume the following ideal chemical reaction (Tieng *et al.*, 1992 ▸),
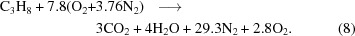
Therefore, the ratio of the molecular density to the electron density for air [7.8(O_2_ + 3.76N_2_); α_air_] is 0.0693; for the mixture of propane and air [C_3_H_8_ + 7.8(O_2_ + 3.76N_2_); α_0_] is 0.0679; and the ratio for gas after the combustion (3CO_2_ + 4H_2_O + 29.3N_2_ + 2.8O_2_; *α*_1_) is 0.0697. Since the system can be expressed by the ideal gas law (Tieng *et al.*, 1992 ▸), we can estimate the temperature distribution from equation (3). Here, α is regarded as a constant value of α_air_ = 0.0693 within an error of 2% in the chemical reaction in (8). The ambient pressure *P* is regarded as a constant. The ambient temperature is 298 K at *x* = 20 mm.

The estimated temperature is shown in Fig. 2(*b*) ▸. The ambient temperature of 298 K is almost maintained in the inner flame region, |*x*| < 4 mm. The temperature increases suddenly at the flame front, reaching 1500 K just outside the flame (|*x*| = 5 mm). The value of 1500 K is confirmed by the thermocouple measurement as shown in Fig. 2(*b*) ▸ and is consistent with the previous report (Tieng *et al.*, 1992 ▸). This suggests the validity of the temperature estimate given by the present Compton scattering analysis and suggests that the chemical reaction is dominated by equation (8). The blue region shown in Fig. 1(*b*) ▸, which is the low electron density region, corresponds to a temperature of 1500 K, although no luminescence is observed in the high-temperature region of 1500 K in Fig. 1(*a*) ▸.

The temperature given by the thermocouple measurement at *x* = 2.5 mm is 1200 K although the temperature measured by the present Compton scattering analysis is the ambient temperature of 298 K as shown in Fig. 2(*b*) ▸. It has been shown that the temperature suddenly decreases to ambient temperature within about a 1 mm distance from the flame front in the inner flame region (Tieng *et al.*, 1992 ▸; Fristrom & Westenberg, 1957 ▸). Therefore the temperature at the position *x* = 2.5 mm, which is 2 mm away from the flame front of *x* = 4.5 mm, should be the ambient temperature of 298 K. This error in the thermocouple measurement may come from thermal conduction from the high-temperature region of 1500 K in the present thermocouple wire. Therefore the present Compton scattering analysis gives accurate position sensitivity in the measurement of the temperature distribution of the flame.

**Figure 3:**
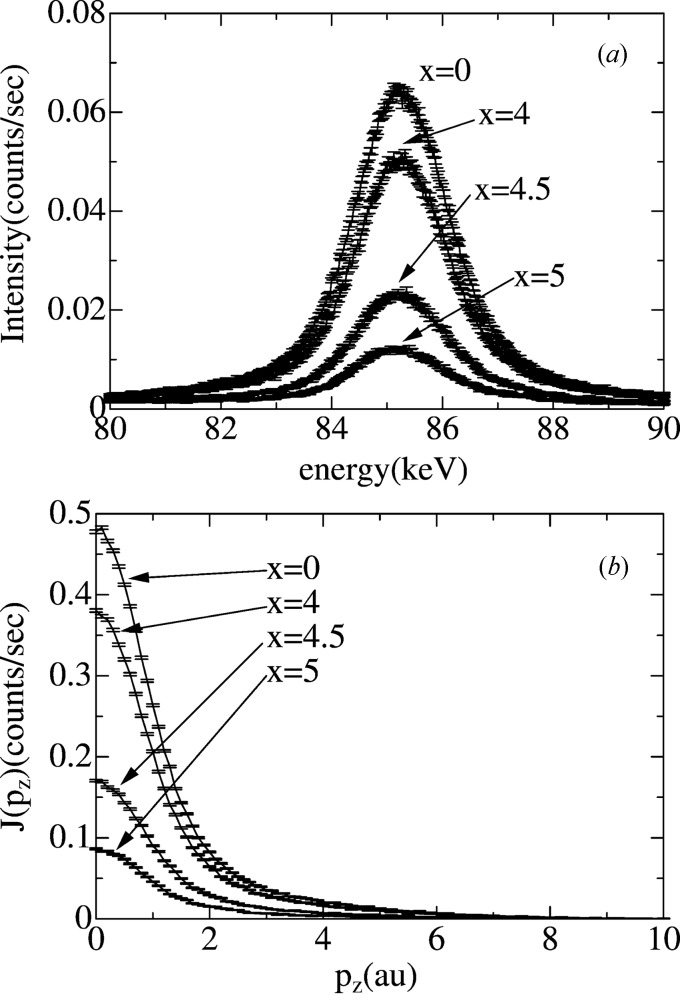
(*a*) Compton-scattered X-ray energy spectra for the flame at the positions *x* = 0 mm (the center of the flame), *x* = 4 mm (just inside the combustion reaction zone), *x* = 4.5 mm (on the combustion reaction zone) and *x* = 5 mm (just outside the combustion reaction zone) shown in Fig. 1(*b*) ▸. Here *y* = 5 mm. (*b*) Electron momentum distributions (Compton profiles) at the positions *x* = 0 mm (the center of the flame), *x* = 4 mm (just inside the combustion reaction zone), *x* = 4.5 mm (on the combustion reaction zone) and *x* = 5 mm (just outside the combustion reaction zone) shown in Fig. 1(*b*) ▸. Here *y* = 5 mm.

Fig. 3(*a*) ▸ shows energy spectra of Compton-scattered X-rays at the position of *y* = 5 mm and *x* = 0 mm, 4 mm, 4.5 mm and 5 mm shown in Figs. 1(*b*) ▸ and 2 ▸. Compton-scattered X-rays have a peak at 85.27 keV, which corresponds to *p*_*z*_ = 0 atomic units (a.u.) in equations (4)–(6). The peak broadening comes from the Doppler shift of X-ray photons scattered by moving electrons in molecules. The intensities of Compton-scattered X-rays, which are defined as the sum of the counts between 80 keV and 90 keV, depend on the position *x*. The intensity decreases with increasing value of *x*, which has been discussed with reference to Figs. 1(*b*) ▸ and 2(*a*) ▸.

The energy spectra shown in Fig. 3(*a*) ▸ can be converted into electron momentum distributions by using equation (7), which are called Compton profiles, as shown by equations (4)–(6). Fig. 3(*b*) ▸ shows Compton profiles, *J*(*p*_*z*_), obtained from Fig. 3(*a*) ▸. The linear background contributions in Fig. 3(*a*) ▸ are removed. The Compton profiles shown in Fig. 3(*b*) ▸ give information on chemical bonding as explained earlier. In order to highlight the effects of chemical bonding, difference Compton profiles, Δ*J*_*x*mm_(*p*_*z*_), are obtained as follows,
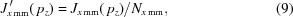


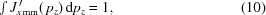


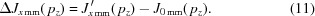
Here *J*_*x*mm_(*p*_*z*_) denotes a Compton profile at *x* mm as shown in Fig. 3(*b*) ▸. A normalization factor, *N*_*x*mm_, is obtained so as to satisfy equation (10). The difference Compton profile Δ*J*_*x*mm_(*p*_*z*_) reflects the difference of chemical bonding between *x* = 0 mm, where the gas is a mixture of air and combustion gas, and *x* mm, where the gas is under combustion reaction.

**Figure 4:**
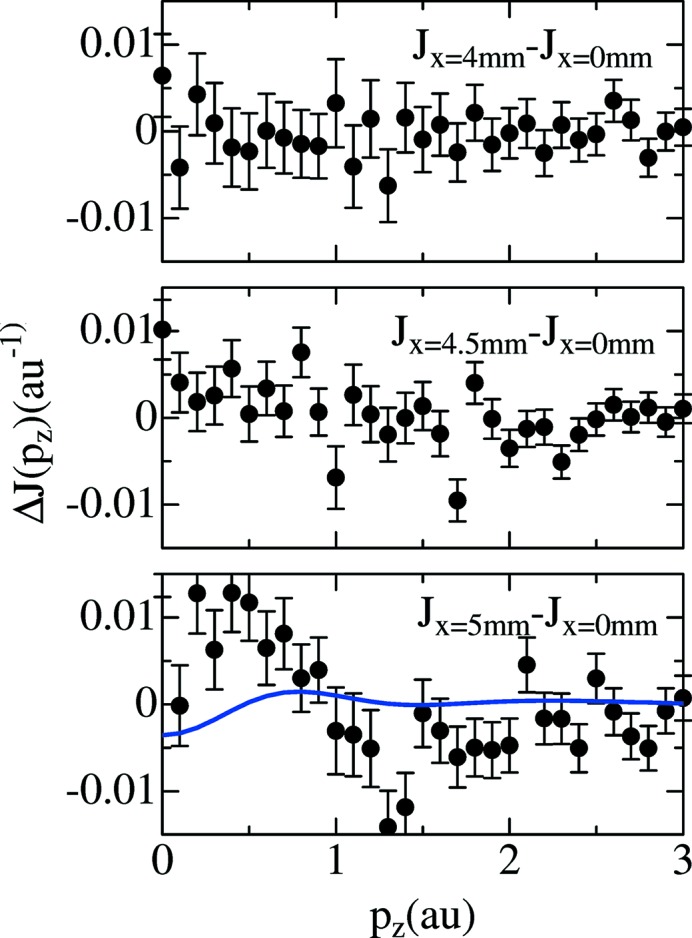
Difference Compton profiles, Δ*J*_*x*mm_(*p*_*z*_), between the position at the center of the flame, *x* = 0 mm, and *x* = 4 mm (just inside the combustion reaction zone), *x* = 4.5 mm (on the combustion reaction zone) and *x* = 5 mm (just outside the combustion reaction zone) shown in Fig. 1(*b*) ▸. Here *y* = 5 mm. A calculated difference Compton profile using *CRYSTAL09* codes assuming the ideal chemical combustion reaction is shown by a solid line.

Fig. 4 ▸ shows Δ*J*_*x*mm_(*p*_*z*_) just inside the combustion reaction zone (*x* = 4 mm), at the combustion reaction zone (*x* = 4.5 mm) and just outside the combustion reaction zone (*x* = 5 mm). Δ*J*_*x*mm_(*p*_*z*_) just inside the combustion reaction zone (*x* = 4 mm) is almost zero within experimental error (see Fig. 4 ▸). This shows that the chemical reaction does not take place. This is consistent with the fact that the ambient temperature of 298 K is almost maintained just inside the combustion reaction zone, |*x*| < 4 mm, as shown in Fig. 2(*b*) ▸. It should be noted that Δ*J*_*x*mm_(*p*_*z*_) in the combustion reaction zone (*x* = 4.5 mm) is almost zero within experimental error as shown in Fig. 4 ▸, although bright luminescence of the combustion reaction zone is observed as shown in Fig. 1(*a*) ▸ and the temperature is 800 K as shown in Fig. 2(*b*) ▸. This suggests that the main type of chemical bonding remains but the combustion reaction, such as a radical luminescence, starts at the combustion reaction zone. Δ*J*_*x*mm_(*p*_*z*_) just outside the combustion reaction zone (*x* = 5 mm) deviates from zero as shown in Fig. 4 ▸. This shows that the main type of chemical bonding, or molecular species, has changed drastically. This means that the combustion reactions complete just outside the combustion reaction zone and high-temperature region of 1500 K with no luminescence extending into the outer region of the combustion reaction zone as a result of the flame gas flow.

The features of Δ*J*_*x*mm_(*p*_*z*_) just outside the combustion reaction zone (*x* = 5 mm) in Fig. 4 ▸, which correspond to a change of chemical bonds, are a dip structure with |*p*_*z*_| < 0.5 a.u., positive values with |*p*_*z*_| < 1 a.u. and negative values with |*p*_*z*_| > 1 a.u. as shown in Fig. 4 ▸. The calculated Δ*J*_5mm_(*p*_*z*_) assuming the ideal chemical reaction in equation (8) by using *CRYSTAL09* (Dovesi *et al.*, 2005 ▸, 2009 ▸) is also shown in Fig. 4 ▸. Although the dip structure with |*p*_*z*_| < 0.5 a.u. seems to be reproduced by the calculation, positive values with |*p*_*z*_| < 1 a.u. and negative values with |*p*_*z*_| > 1 a.u. are not reproduced. Since the ideal chemical reaction in equation (8) dominates the system as explained by Fig. 2(*b*) ▸, positive values with |*p*_*z*_| < 1 a.u. and negative values with |*p*_*z*_| > 1 a.u. can be attributed to partially generated radical species such as OH^*^ and atomic H (Beduneau *et al.*, 2009 ▸).

**Figure 5:**
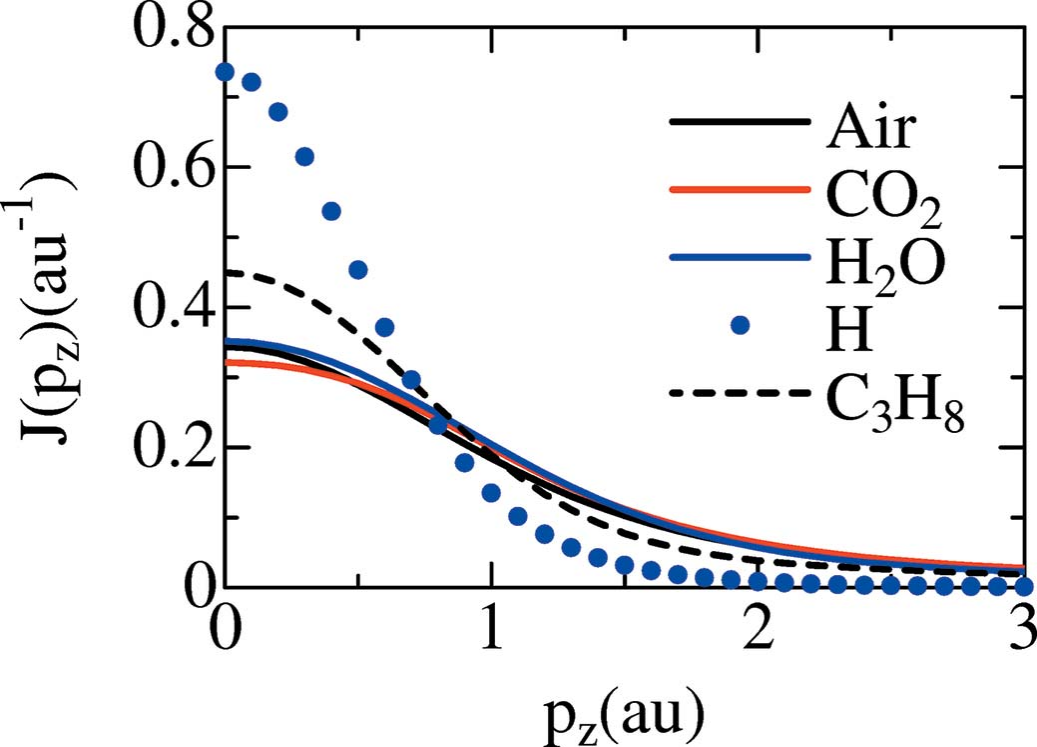
Calculated Compton profiles using *CRYSTAL09* for air (O_2_ + 3.76N_2_) (black solid line), CO_2_ (red solid line), H_2_O (blue solid line), C_3_H_8_ (black dashed line) and Hartree–Fock calculation (Biggs *et al.*, 1975 ▸) for atomic H (blue dots). The Compton profiles are normalized using equations (9) and (10) (see text).

Fig. 5 ▸ shows calculated Compton profiles using *CRYSTAL09* for air (O_2_ + 3.76N_2_), CO_2_, H_2_O, C_3_H_8_ and Hartree–Fock calculation (Biggs *et al.*, 1975 ▸) for atomic H. The Compton profiles shown in Fig. 5 ▸ are normalized using equations (9) and (10). The CO_2_, which has smaller *J*(*p*_*z*_) values with |*p*_*z*_| < 0.5 a.u. than that of air, is ascribed to the dip structure with |*p*_*z*_| < 0.5 a.u. The C_3_H_8_ and the atomic H have larger *J*(*p*_*z*_) values with |*p*_*z*_| < 1 a.u. and lower values with |*p*_*z*_| > 1 a.u. than that of air. Because the combustion is complete and the temperature increases to 1500 K just out of the flame front as shown in Fig. 2 ▸, there seems to be no uncombusted C_3_H_8_ (Tieng *et al.*, 1992 ▸; Fristrom & Westenberg, 1957 ▸). Thus the positive values with |*p*_*z*_| < 1 a.u. and negative values with |*p*_*z*_| > 1 a.u. are attributed to partially generated radical species such as atomic H accompanied by OH^*^ radical. Therefore it is suggested that partially generated radical species such as OH^*^ and atomic H contribute to the region just outside the combustion reaction zone (*x* = 5 mm) in Fig. 4 ▸ although the ideal chemical reaction in equation (8) dominates.

In conclusion, the intensity of Compton-scattered X-rays from the flame of a combustion gas has been measured. We have evaluated the local temperature from the X-ray intensity, and the temperature distribution across the flame is obtained with accurate position sensitivity. The energy spectra of Compton-scattered X-rays have shown a small but significant variation across the combustion reaction zone, which reflects partially generated radical species such as OH^*^ and atomic H by the combustion reaction. Since it uses high-energy X-rays with high-penetrating power, it will be possible that the Compton scattering technique can be developed as an *in situ* and *operando* measurement for probing the inside of a combustion reaction.
